# Supplementary material for: Gram-Level Production of Balanol through Regulatory Pathway and Medium Optimization in Herb Fungus Tolypocladium ophioglossoides
Source: J Fungi (Basel). 2022 May 16;8(5):510. doi: 10.3390/jof8050510 (PMC9143294; doi:10.3390/jof8050510)
Supplement: Supplementary file 1 [file jof-08-00510-s001.zip › jof-1701558-supplementary.pdf]

Supplementary Material

Gram-Level Production of Balanol through Regulatory Pathway and Medium Optimization in Herb Fungus *Tolypocladium ophioglossoides*

Rui-Qi Li [1,2], Xiang Liu [1,2], Min Zhang [1,2], Wei-Qun Xu [1], Yong-Quan Li [2] and Xin-Ai Chen [1,2,*]

Table S1. Primers used in this study.

Primer	Sequence
blnR-F	tgatatcggatccgaattcATGGCCTCAGAAACCCGCGCC
blnR-R^{DBD}	aagcttgtcgacggagctcGGACGTCGTCGACCCGAAAGCC
blnR-R^{180aa}	agcttgtcgacggagctcATCAACGGGTGCATTCTGGTCG
blnR-R^{292aa}	agcttgtcgacggagctcGGACGAAGCGTCACCACTTTCA
blnR-R^{361aa}	agcttgtcgacggagctcGTCAGCACTGTCCAAGGTGTCG
AbaA-qRT-F	ACATGTCGGCTTTGGACAACTG
AbaA-qRT-R	CACATGGCTGTCCATCGCATC
BrlA-qRT-R	CTCTTGTTCGATCACTGGCACAG
BrlA-qRT-F	GTGCCACAAGGCTTTCCGTC
FlbC-qRT-R	GCGGTGGACTTTTCGGTGTC
FlbC-qRT-F	GACTCATGGGCTCCCAAATGC
FlbD-qRT-F	GACCAGCCACTGCCTAGCAG
FlbD-qRT-R	CAGCCTTCTCATATCCATCCGC
FluG-qRT-F	GAGGAGCCAGCATCGCTTTC
FluG-qRT-R	GTCCATCGACTCGAGCAATTC
FAM-P1F	AATAAAGGAGGCGCGACGG
P1-R	CAACTGTGCCTGTTGGGTCTG
FAM-P2F	CATCGTGCCAGCTGAAGCG
P2-F	TAACATCCGGTGGTCGGAGTTTG
FAM-P3F	TGCCATCTGGACGAAGTCGG
P3-R	GAGGGCTTCATCGGACCCGA
FAM-P4F	GCATTGAGTCCCCAGAGCCA
P4-R	CTGGCCGGCTCGACATAATACT
FAM-P5F	GGATCCAGAAGAGGCTTGCAG
P5-R	TCATTTGACGGGCCGGATTTGC
FAM-P6F	AAGGCAATGGCGGAATGCAC
P6-R	GGCAAGGGCAATCTGAGAAGC
FAM-P7F	TGCCGTTCCACAATTTCCGGT
P7-R	GGTGGCAAATATGTGCGGTTGT
FAM-PA8F	CGCGGGTTTCTGAGGCCATA
P8-R	GCTTCGCGGTGTCGTTGGA

FAM-P9F	CCAACGACTGAGCCAGTGAG
P9-R	TTGGACAGCCGGCGTCATT
FAM-P10F	TCCAAGAGCTCCTCCAGGCT
P10-R	ATGATGCTGCCCATGATGCTGC
FAM-P11F	GCAACTTGTGAACGGGGATCG
P11-R	CCGAGTCGTGCGACTGCTTA
FAM-P12F	GACGATGGCCTTGAGGCGTA
P12-R	CCGACACCCTGTTCGAATTGG

Table S2. The scheme and experimental results of response surface design.

**Run**	**A-Sucrose (g/L)**	**B-Polypeptone (g/L）**	**C-pH**	**Balanol（mg/L）**
**1**	−1(90)	1(15)	0(4.5)	672.46
**2**	−1	0(10)	−1(4)	509.31
**3**	1(120)	0	1(5)	1013.23
**4**	0(105)	0	0	1785.25
**5**	0	0	0	1593.02
**6**	−1	0	1	1199.81
**7**	0	0	0	1947.94
**8**	1	1	0	920.087
**9**	0	0	0	1880.35
**10**	0	1	−1	468.53
**11**	0	−1(5)	−1	852.54
**12**	0	1	1	1285.83
**13**	0	−1	1	1453.14
**14**	1	−1	0	841.99
**15**	−1	−1	0	1024.14
**16**	1	0	−1	458.00
**17**	0	0	0	1867.72
